# Cost of Sickness Absenteeism during Seasonal Influenza Outbreaks of Medium Intensity among Health Care Workers

**DOI:** 10.3390/ijerph16050747

**Published:** 2019-03-01

**Authors:** Maria Michela Gianino, Gianfranco Politano, Antonio Scarmozzino, Michela Stillo, Viola Amprino, Stefano Di Carlo, Alfredo Benso, Carla Maria Zotti

**Affiliations:** 1 Department of Public Health Sciences and Pediatrics, Università di Torino, via Santena 5 bis, 10126 Torino, Italy; michelasti@gmail.com (M.S.); vamprino@aslto3.piemonte.it (V.A.); carla.zotti@unito.it (C.M.Z.); 2 Department of Control and Computer Engineering, Politecnico di Torino, Corso Duca degli Abruzzi, 24, 10129 Torino, Italy; gianfranco.politano@polito.it (G.P.); stefano.dicarlo@polito.it (S.D.C.); alfredo.benso@polito.it (A.B.); 3 AOU Città della salute e della Scienza, Torino, corso Bramante 88/90, 10126 Torino, Italy; ascarmozzino@cittadellasalute.to.it

**Keywords:** costs, absenteeism, healthcare workers, seasonal influenza epidemics, medium intensity

## Abstract

This study aims to estimate the economic costs of sickness absenteeism of health care workers in a large Italian teaching hospital during the seasonal flu periods. A retrospective observational study was performed. The excess data of hospital’s sickness absenteeism during three seasonal influenza periods (2010/2011; 2011/2012; 2012/2013) came from a previous study. The cost of sickness absenteeism was calculated for six job categories: medical doctor, technical executive (i.e., pharmacists); nurses and allied health professionals (i.e., radiographer), other executives (i.e., engineer), non-medical support staff, and administrative staff, and for four age ranges: <39, 40–49, 50–59, and >59 years. An average of 5401 employees working each year were under study. There were over 11,100 working days/year lost associated with an influenza period in Italy, the costs associated were approximately 1.7 million euros, and the average work loss was valued at € 327/person. The major shares of cost appeared related to nurses and allied health professionals (45% of total costs). The highest costs for working days lost were reported in the 40–49 age range, accounting for 37% of total costs. Due to the substantial economic burden of sickness absenteeism, there are clear benefits to be gained from the effective prevention of the influenza.

## 1. Introduction

Epidemic influenza is a contagious disease that affects people of all ages and imposes substantial burdens on healthcare systems. The World Health Organization (WHO) estimates that 5–15% of the population is affected during each annual influenza epidemic, which entails 3–5 million cases of severe illness worldwide each year. The influenza epidemic also impacts the working population and a systematic review and meta-analysis of influenza incidence in health care workers (HCWs) and other healthy adults suggested that HCWs are at higher risk for influenza infection as compared to healthy adults working in non-healthcare settings [1].

Influenza can contribute to significant absenteeism during annual epidemics or occasional pandemics. Although by few studies, the assessment of the impact of influenza on work has been undertaken in the health care sector. An epidemic of influenza A in Winnipeg resulted in an almost two-fold increase in absenteeism by nurses and support personnel in comparison with reminded period of the year 1980/1981 [2]. In British Columbia, a retrospective cohort study of full-time HCW that worked prior to and during the 2012/2013 influenza season showed an absenteeism increase of 0.69 h/per 100 scheduled hours from the pre influenza to the influenza season [3]. In 2001, Sartor described an influenza outbreak that occurred in an internal medicine unit of an acute care hospital in France [4]. During the study period (28 February to 6 March, 1999), the absenteeism in staff members amounted to 14 days of sick leave, an average of 2.8 days lost per episode, with the absence days being split between physicians (1 day), nursing staff (6 days), and non-nursing staff (7 days). In Japan, a study was conducted to assess absenteeism in health-care workers of Gifu Red Cross Hospital during the 2002/2003 influenza season. Among vaccine recipients, the number of influenza infections was 3.4 per 100 subjects, compared with 8.5 per 100 subjects in nonrecipients (*p* = 0.034). The number of days absent from work was 9.5 per 100 subjects for vaccine recipients, compared with 15.1 per 100 subjects for non-recipients (*p* = 0.0003) [5]. In 2008, another study conducted in Taiwan reported that the average number of workdays lost due to ILI (Influenza-like illness) was 1.09 in vaccinated HCWs and 1.5 in non-vaccinated ones [6].

Seasonal influenza is also a major economic burden. It can result in increased healthcare system costs because of increase on the usage of medical resources, such as prescribed medication, laboratory investigations, outpatient services, and hospitalizations, required to treat patients and related complications, with economic consequences for the healthcare system [7,8]. Moreover, seasonal influenza can result in increased society financial burden because of associated cost of the loss of working time and then of productivity [9,10,11]. Indeed, the absenteeism impacts directly on individuals, teams, and the organization as a whole, putting pressure on productivity. Simply put, if someone works less, they are likely to be less productive, as missed work time increases, and workers may be left making up for the work not performed by employees who are absent. Even supervisor productivity is impacted because they must spend more time dealing with absences and preparing for and adjusting workflow to guarantee the provision of healthcare services.

Although the studies above have attempted to quantify the impact of influenza upon HCWs in terms of lost working days associated with an episode of influenza, the socio-economic consequences of absence due to sickness among HCWs has been poorly documented. Information on costs of absenteeism may be useful to initiate preventive and protective interventions, targeted also on the most absent worker categories, able to guarantee delivering healthcare services. In addition, these interventions may reduce the negative impact of health problems of HCWs on work efficiency, work efficiency, quality of patient care, and patient safety.

The aim of this study is to estimate the economic costs of sickness absenteeism in a large Italian hospital during the seasonal flu period. A further aim was to analyze the distribution of the sick leave during the influenza.

## 2. Methods

### 2.1. Design

An observational and cross-sectional study was performed. The study protocol was approved by the Directorate-General of AOU Città della salute e della Scienza (26 November 2013).

In this study, we analyzed the number of days of paid sick leave among HCWs of “AOU Città della salute e della Scienza”. It is a complex of four interconnected teaching hospitals with more than 2500 beds in Turin. We studied Molinette, one of the four hospitals. In this study, without sampling, we analyzed the absenteeism data that included all HCWs of Molinette which has approximately 5500 workers (approximately 45% of the center’s employees).

### 2.2. Absenteeism Data Source

The absenteeism data and data on the influenza vaccination coverage in the hospital, have been widely described in a previous study [12]. Briefly, the absenteeism data were obtained from the hospital’s Personal Unit Database, and from the Occupational Health Unit of Molinette Hospital we obtained data on influenza vaccination for each employee; the data were directly available from Directorate-General of AOU, anonymously.

The study focuses on the “sporadic absence”, defined in the previous study above cited [12] as unplanned sickness absence, due to any cause certificated by medical practitioner and paid. “Sporadic absence” doesn’t include planned absences (e.g., pregnancy, absences for traumas, chronic therapies, etc.).

Due to the Italian policy regarding absenteeism records in the workplace, it is not compulsory to note the medical diagnosis reported on the sickness sporadic absence certificate issued by the medical practitioner; consequentially, our dataset includes not only Influenza-like illness (ILI)-related or acute respiratory infection (ARI)-related absences, but all sporadic absences.

The division of the sick leave data from different years into three “influenza epidemic periods” and three “non-epidemic periods” was made. “Influenza epidemic periods” were defined as week 42nd of one year to week 17th of the following year (2010/2011, 2011/2012, 2012/2013) and “non-epidemic periods” which started at week 18 and terminated at week 41 and used as baseline data [13,14]. The previous study [12] showed that total days of absenteeism in epidemic and remaining period of the years were the following (Table 1):

### 2.3. Economic Data

Measuring absenteeism costs has been performed through “Human capital method”. The human capital approach places a monetary value on absenteeism as the lost value of economic productivity due to ill health. The present value of wages is used to evaluate the lost value of economic productivity.

The daily cost of absenteeism was calculated as follows: Annual gross wage for each category/Total number of working days in each year.

From the Italian Ministry of Health database, we obtained the annual gross salary (wage) for each job category: Medical doctor; Technical executive (pharmacists, dieticians, chemists, and the like), Nurses and allied health professionals (radiographers, therapists, laboratory technicians, and the like); Other executives (engineers, lawyers, analysts, statistical, administrative); Non-medical support staff (ward assistants, cleaning staff); Administrative staff.

From the collective labor agreement, we obtained the number of non-working days, public holidays, and holidays. The total number of working days was 264 and excludes non-working days, public holidays, and holidays. The costs of working days for each job category were the following: Medical doctor, € 412; Technical executive (pharmacists, dieticians, chemists, and the like), € 402; Nurses and allied health professionals (radiographers, therapists, laboratory technicians, and the like), € 164; Other executive (engineers, lawyers, analysts, statistical, administrative), € 387; Non-medical support staff (ward assistants, cleaning staff), € 132; Administrative staff, € 136.

The Italian Ministry of Health database does not provide age-specific costs, so the cost of working days for each job category is a weighted average cost because it has been calculated starting from the sum of the wages of workers of all age classes (and consequently wage levels) in each job category.

Costs of lost workdays were calculated by multiplying the estimated number of workdays missed by the weighted average daily wage for each category.

All costs were expressed at the 2013 costs level.

### 2.4. Statistical Analysis

#### 2.4.1. Data Preprocessing

As detailed in Gianino [12], we fed the pipeline with absenteeism data containing, for each employee, several attributes used for stratification (e.g., position, contact with patients, sex) and weekly days of absenteeism, up to 7 days per week over 52 weeks (i.e., 364 days). The strata frequency distribution and the trend in the flu absenteeism over the three-year timespan is computed for each attribute, and the results have been represented in terms of days lost per person due to illness. This helped to efficiently compare values belonging to multiple heterogeneous populations.

#### 2.4.2. Data Analysis

Pre-processed data have been analyzed as follows: we computed (i) the frequency distribution within the available classes for each attribute, and (ii) the average and cumulative rates of absenteeism during epidemic and non-epidemic periods and the excess absenteeism during epidemic periods (working days lost per person per year) stratified for job category and age. We used epiR [15] and meta [16] packages to perform a risk analysis able to determine the average risk of absenteeism (i.e., risk to lose a day of work) due to the influenza period.

On top of computed risk analysis, we derived a weighted attributable risk in order to guarantee a greater strength of the results. Incidental risks (IR) computed through the risk analysis have been used to quantify the extra days lost due to flu (EDLF) according to the following formula:

EDLF = EPD × IR_epd_ − NEPD × IR_nepd_,

where EPD and NEPD represent the number of days for epidemic period (EPD = 196 days) and non-epidemic period (NEPD = 168 days) and IR represents the incidental risk for epidemic period (IR_epd_) and non-epidemic period (IR_nepd_).

EDLF so far computed offers a more meaningful and clear quantification by transforming the percentage (i.e., incidental risk) of absenteeism in the underlying amount of days lost per person (see Table 2). This transformation results in easier to compare figures, moreover, since the timespan varies between epidemic and non-epidemic periods, and it is important to stress that EDLF is a weighted measure, which takes in account differences between timeframes (i.e., EPD, NEPD). The IRs (i.e., percentage representing the risk of absenteeism in epidemic and non-epidemic days) need in fact to be multiplied for the proper timeframe amount of days (i.e., EPD, NEPD) in order to correctly represent their underlying risk values in terms of days lost, which eventually result in more comparable figures.

Furthermore, in order to properly deal with variations in hospital population over the three-year timespan, as already discussed in [12], we resorted to the Mantel–Haenszel (MH) method [17], which generates a fixed-effect model able to estimate the association between an exposure and an outcome by taking into account confounding in stratified categorical data. This is particularly helpful in order to obtain a more robust averaged evaluation over the entire timespan of the dynamic hospital population (i.e., three years), and obtain a significance score (MH test) able to confirm the overall homogeneity of effects in the analysis. After MH confirmation, each strata-associated confusion matrix was further tested against Fisher’s Exact Test for Count Data in order to assess the overall significance with its *p*-value.

#### 2.4.3. Output

The final output of the pipeline is a file containing stratified and cumulative costs related to working days lost per person per year in epidemic periods. The costs were computed by multiplying the strata/cumulative average EDLF score (see Section 2.4.2) and the personnel costs discussed in Section 2.1. No specific methods for sensitivity analysis were performed on the dataset since it was monolitically built by the hospital datacenter, which in turn made the whole dataset consistent and did not provide any quality level on which restrict the data included. So, the analysis was performed on the entire dataset.

## 3. Results

Within the hospital studied, an average of 5401 employees worked each year (5544, 5369, and 5291 in 2010/2011, 2011/2012 and 2012/2013, respectively, and the reduction in the number of workers was affected by retirement). Of these employees, approximately 73% were female.

The categories of ‘Nurses and allied health professionals’ and ‘Nonmedical support staff’ were the largest, with approximately 2500 and 1300 workers, respectively. The least numerous category was the ‘Other Executive’ category, with 30 employees (Table 2).

A percentage of 72% of workers were aged 40–59 (38% = 40–49 years of age, and 34% = 50–59 years of age), while only 6% were aged >59 years.

The highest percentage of workers aged <39 years was observed among Nurses and allied health professionals, and the lowest percentage among Other executives (i.e., 0%).

The lowest percentage of workers aged >59 years was observed among Nurses and allied health professionals, and the highest percentage in the Technical executive job category.

The vaccinated HCWs was below 3% during the selected three-year period, and consequently the inclusion or exclusion of vaccinated workers have not significantly impacted on the trend in absenteeism.

As already shown in the previous study [12], the average level of absenteeism during the epidemic period increased for all job categories (Table 3). In comparison with the other job categories, the absolute increases were highest for Non-medical support staff (+3.4 days/person), Administrative staff (+2.5 days/person), and Nurses and Allied health professionals (+1.95 days/person).

Table 3 also shows the significant increase in sick leave during the seasonal influenza epidemic for all age ranges. In comparison with the other age ranges, the absolute increase was lowest in >59 age range (+1.95 days/person).

The workers in the 50–59 and >59 age ranges experienced a relative increase lower than those for younger employees. These increases were 57% and 61%, respectively. The workers in the 50–59 and >59 age ranges exhibited higher absenteeism in non-epidemic periods in comparison with the other workers (more than 3.4 days/person vs. less than 3.0 days/person).

The <39 age range showed the highest increase in absenteeism among Medical doctors (+1.45 days/person), Nurses and allied health professionals (+2.07 days/person), and Non-medical support staff (+3.74 days/person). The 40–49 age range showed the highest absenteeism increase among Administrative staff (+2.65 days/person), and the 50–59 age range among Technical executives.

Since there were so few Other executives, it was not possible to calculate the increase by age in that category.

Table 4 shows the costs from the increase in sick leave during the epidemics for all job categories.

In terms of absenteeism from work, the overall total cost was an estimated at € 1,763,683.

For the distribution of costs per job category, the major shares of cost appear related to the categories of Nurses and Allied health professionals (45% of total costs) and Nonmedical support staff (32% of total costs). A minor share of cost was related to Other executives (0.2% of total costs).

For the distribution of costs for age ranges, the highest costs for working days lost among workers are reported in the 40–49 and 50–59 age ranges, accounting for 37% and 34% of total costs, respectively.

## 4. Discussion

This study demonstrated that epidemic periods substantially affects HCW absenteeism, with significantly higher sick leave than during non-epidemic periods. There were over 11,100 working days lost per year associated with sickness absenteeism during seasonal influenza periods in Italy. The costs associated with this increase of working days lost were approximately 1.7 million euros, and the average paid sick leave was valued at € 327 per person.

These results indicated that an influenza epidemic accounts for thousands of days lost from work and causes substantial economic losses via HCW sick days. They also confirmed, with focus on HCWs, previous studies conducted around the world that estimate the indirect cost due to ILI in the work place in terms of absences from work [10,18,19,20,21]. This economic burden is high, but it is coherent with that total days of absenteeism in epidemic and remaining period of the years reported in the methods section.

A contribution to this level of cost was offered both by the multiple job categories and by the different composition by age ranges of workers.

The Nurses and allied health professionals and Nonmedical support staff categories, along with the 40–49 and 50–59 age ranges, were responsible for most of the costs of absenteeism. This result may be explained because Nurses and allied health professionals and Nonmedical support staff were the larger groups (46% and 23%, respectively, of the total sample) and showed the highest increase of sick leave (+1.95 and +3.4 days/person lost, respectively), even if the working daily costs were lower than other job categories (€ 164 and € 132, respectively). In contrast, the lower costs of absenteeism by Other executives may be explained because this category showed a low increase in sick leave (+0.36 days/person lost), and it was numerically not significant (30 workers).

Although many studies of sickness-related absence have been conducted among healthcare personnel, most of these have concentrated on all causes of absence [22,23]. However, these analyses are of value because they showed that rates of absence are related to socioeconomic status, they can be generalized to absenteeism attributable to influenza epidemics, and they support our findings.

Although the excess of sick leave was similar across the various age groups, the 40–49 age range explains most of the costs. This result was affected by the weight of the cost of lost days from the category of Nurses and allied health professionals, because that category has the most employees in that age range. It is followed by the 50–59 age range, for which the category of Non-medical support staff played an important role. This category included many workers with a high increase in absenteeism during the epidemic period. In contrast, the absenteeism costs from the workers 59 years old and over were low. The lowest values of increased absenteeism occur in the over 59 age range and the Medical doctors (+0.14 days/person and Technical executive (−0.4 days/person) categories, which may suggest that older workers exhibit a lower absence rate because of a higher job commitment and a better person–organization fit that emerges over time [24,25,26].

This study has some limitations. Firstly, our study used sporadic absence, defined as an unplanned sickness absence due to any cause certified by Medical Practitioners, which is not only influenza-related. We should consider that we are working on an excess of absenteeism, characteristic of a certain period of the year, given the premise that non-flu related absenteeism is uniformly distributed through the year. However, in according to other studies, the circulation of different types of viruses can confound the clinical diagnosis of Influenza-Like Illness and lead to an overestimation of acute respiratory illness [27,28]; only the viral-culture-confirmed sick leave may lead to more robust estimates of work absence due to seasonal influenza epidemics. Secondly, costs were applied to the number of missed work days using the hospital’s human resources figures. It may be that the actual loss to Molinette hospital was far less than the estimated indirect costs of loss of potential productivity calculated by this human capital approach [29,30,31]. The exact relationship between short-term absence and loss of productivity depends on the absentee’s profession, the type of organization, and the production process [31,32]. Thus, an Administrative staff worker may make up for lost time on return to work, and Nurses and allied health professionals may cover for colleagues during absences. Finally, the daily cost of absenteeism was valued using the weighted average cost of a working day for each job category, regardless of age. This may mean a different distribution of the economic burden of absenteeism among the different age groups: lower in younger age groups and higher in older age groups. Nevertheless, using age-specific costs does not affect the conclusions, given the age classes distribution of workers in every job category and of incremental days lost. Indeed, the weighted average cost of the job category is conditioned by the cost of the highest density age class; the highest density age class, given the distribution of the lost days for every age class, is the one that shows the greatest number of lost workdays.

## 5. Conclusions

Due to the substantial economic burden of absenteeism during influenza epidemic periods, there are clear benefits to be gained from the effective prevention of the flu. A range of options are available, including seasonal influenza vaccination, which has been recommended for HCWs in almost all European countries for many years. However, despite efforts to encourage healthcare workers to get immunized against influenza, vaccination levels remain insufficient in Europe: available data demonstrate that after thirty years of official recommendations, the immunization rate among HCWs rarely exceeds 30%–40% [33]. In Italy, the general population achieves only a 14% coverage rate overall, with rates of 20%–25% in adults affected by chronic medical conditions and not more than 15% in HCWs; in “AOU Città della Salute e della Scienza” during the three seasons studied, the rate for HCWs was below 3%.

As a recent study has shown [34], mandatory vaccination improves compliance to vaccination and reduces the absenteeism of HCWs, but Italy has not made a decision about a mandatory immunisation for HCWs at the moment. However, the scientific societies and the HCWs’ professional associations agree on the necessity to improve the approach to vaccination [35] and the health facilities enforce upon themselves to improve the flu vaccine coverage.

While waiting for the mandatory vaccination, the “AOU Città della Salute e della Scienza” and other national health service trusts should implement a human resources policy that improves the approach to immunization and empowers the HCWs on the implications of absenteeism, through training and a rewarding system that supports these goals. Training for HCWs must develop awareness that it is also in the interests of both employees and the trusts that sickness absence is managed and minimized.

The authors also hope that this preliminary estimate of the economic cost of working days may encourage the application of models evaluating the economic implications of vaccine intervention for the HCW population. The difficulty of building these models stems from several reasons: the heterogeneity of the information available; the cost–benefit analysis data are sometimes biased because the studied population is already in a vaccination programme [36]; insufficient epidemiological and economic data about work absenteeism are available [3]; the studied season or the population chosen highlighted little benefit from vaccination [18]. However, cost–benefit analyses have assessed [29,37] that a net savings per person would be most impacted by the lost work productivity, and absenteeism represents approximately 80% of the costs averted by vaccination.

In conclusion, the future research agenda on the subject could concern a comparison on absenteeism in epidemic periods with different intensity (low, medium, high epidemic) to estimate the different consequences on absenteeism and costs, make pre-post studies that evaluate the impact of vaccination coverage among HCWs, and undertake research on efficacy of human resource policies on reductions in sickness absence during seasonal flu periods.

## Figures and Tables

**Table 1:** Absenteism days in epidemic and remaining period of the years.

Seasonal Influenza Periods	Absenteeism Epidemic Periods	Absenteeism in Remaining Period of the Years
**2010/2011**	27,212	16,796
**2011/2012**	25,613	15,035
**2012/2013**	29,226	16,722

**Table 2:** Job category and age range distributions of employees (average over three years).

Job Category	Age Classes				
	<39	40–49	50–59	>59	All Ages
Medical doctor	112	225	356	97	791
Technical executive	8	23	52	17	100
Nurses and allied health professionals	853	1045	542	45	2485
Other executives	0	8	19	3	30
Nonmedical support staff	142	388	604	130	1265
Administrative staff	92	338	267	34	731
All job categories	1207	2027	1841	326	5401

Medical doctor: physicians, radiologists and the like; Technical executive: pharmacists, dieticians, biologists, chemists, and the like; Nurses and allied health professionals: radiographers, therapists, laboratory technicians, and the like; Other executives: engineers, lawyers, analysts, statistical, administrative; Nonmedical support staff: ward assistants, cleaning staff.

**Table 3:** Excess of absenteeism during epidemic periods for age ranges and job categories (working days lost per person per year).

Job Category	Age					Mean Levels of Absenteeism Epidemic Period—All Ages (CI 95%)	Mean Levels of Absenteeism in Remaining Period of The Years—All Ages (CI 95%)
	<39	40–49	50–59	>59	All Ages		
	Incremental days lost year/person (CI 95%)						
Medical doctor	1.45 *	0.61 *	0.42 *	0.14	0.45 *	1.04	0.58
	(1.23–1.67)	(0.51–0.71)	(0.35–0.49)	(0.07–0.21)	(0.4–0.5)	(0.97–1.11)	(0.53–0.63)
Technical executive	0.4	0.82	1.2 *	−0.4	0.92 *	1.91	0.98
	(−0.04–0.84)	(0.45–1.19)	(0.9–1.5)	(−0.1–0.7)	(0.73–1.11)	(1.64–2.18)	(0.79–1.17)
Nurses and Allied health professionals	2.07 *	1.79 *	1.92 *	1.92	1.95 *	4.7	2.75
	(1.97–2.17)	(1.71–1.87)	(1.8–2.04)	(1.52–2.32)	(1.9–2.0)	(4.61–4.79)	(2.68–2.82)
Others executive					0.36 **	0.91	0.56
					(0.15–0.57)	(0.57–1.25)	(0.29–0.83)
Nonmedical support staff	3.74 *	3.05 *	3.52 *	3.59 *	3.4 *	8.57	5.17
	(3.42–4.06)	(2.88–3.22)	(3.37–3.67)	(3.26–3.92)	(3.3–3.5)	(8.41–8.73)	(5.04–5.3)
Administrative staff	1.37 *	2.65 *	1.9 *	1.23	2.15 *	5.22	3.07
	(1.13–1.61)	(2.48–2.82)	(1.73–2.07)	(0.86–1.6)	2.94–3.2)	(5.05–5.39)	(2.94–3.2)
All job categories	2.05 *	2.04 *	2.12 *	1.95 *			
	(1.97–2.13)	(1.98–2.1)	(2.05–2.19)	(1.8–2.1)			
	Mean levels of absenteeism in Remaining period of the years (CI 95%)						
All job categories	2.59	2.82	3.46	3.44			
	(2.5–2.68)	(2.75–2.89)	(3.38–3.54)	(3.24–3.64)			
	Mean levels of absenteeism Epidemic periods (CI 95%)						
All job categories	4.64	4.85	5.58	5.39			
	(4.52–4.76)	(4.75–4.95)	(5.47–5.69)	(5.14–5.64)			

* *p* value *p* < 0.01; ** *p* value *p* < 0.05. Medical doctor: physicians, radiologists, and the like; Technical executive: pharmacists, dieticians, biologists, chemists, and the like; Nurses and allied health professionals: radiographers, therapists, laboratory technicians, and the like; Other executives: engineers, lawyers, analysts, statistical, administrative; Nonmedical support staff: ward assistants, cleaning staff.

**Table 4:** Costs for days lost/year in epidemic periods (Euros).

Job Category	Age Classes				
	<39	40–49	50–59	>59	All Ages
Medical doctor	66,858	56,587	61,613	5591	146,479
Technical executive	1341	7473	25,244	2681	36,984
Nurses and allied health professionals	289,464	306,867	170,769	14,170	794,809
Other executives					4180
Nonmedical support staff	70,265	156,342	280,489	61,761	567,584
Administrative staff	17,141	121,696	68,993	5688	213,648
All job categories	445,070	648,965	607,108	84,528	1,763,683

Medical doctor: physicians, radiologists and the like; Technical executive: pharmacists, dieticians, biologists, chemists, and the like; Nurses and allied health professionals: Radiographers, Therapists, Laboratory technicians and the like; Other executives: engineer, lawyer, analyst, statistical, administrative; Nonmedical support staff: ward assistants, cleaning staff.
